# Supplementary material for: The Systems Biology Research Tool: evolvable open-source software
Source: BMC Syst Biol. 2008 Jun 29;2:55. doi: 10.1186/1752-0509-2-55 (PMC2446383; doi:10.1186/1752-0509-2-55)
Supplement: Additional file 1 — SBRT Archive. An archive of the current version of the Systems Biology Research Tool. [file 1752-0509-2-55-S1.zip › sbrt-1.4.0/doc/developers_guide/api/sbrt/kernel/math/algebra/package-use.html]

Uses of Package sbrt.kernel.math.algebra


|  |  |  |
| --- | --- | --- |
| **Overview** \| **Package** \| Class \| **Use** \| **Tree** \| **Deprecated** \| **Index** \| **Help** | |  |
| PREV   NEXT | **FRAMES**    **NO FRAMES**     **All Classes** |  |


---


## **Uses of Package sbrt.kernel.math.algebra**

| Packages that use sbrt.kernel.math.algebra | |
| --- | --- |
| **sbrt.ext.mma.algebra** | Provides classes that allow communication with Mathematica for the purposes of algebra. |
| **sbrt.kernel.fba** | Provides classes and interfaces for creating objects relevant to Flux Balance Analysis. |
| **sbrt.kernel.fba.comp** | Provides classes and interfaces for performing Flux Balance Analysis computations. |
| **sbrt.kernel.fba.proc** | Provides classes and interfaces for executing Flux Balance Analysis processes. |
| **sbrt.kernel.math.algebra** | Provides classes and interfaces for creating objects relevant to algebra. |
| **sbrt.kernel.math.algebra.proc** | Provides classes and interfaces for executing algebra-related processes. |
| **sbrt.kernel.math.geom** | Provides classes and interfaces for creating objects relevant to computational geometry. |
| **sbrt.shell.io** | Provides classes and interfaces for reading and writing files. |
| **sbrt.shell.mng.fba** | Provides classes and interfaces for creating Flux Balance Analysis process managers. |
| **sbrt.shell.text.math** | Provides classes and interfaces for defining formats for mathematical objects. |

Classes in sbrt.kernel.math.algebra used by sbrt.ext.mma.algebra

****LinearSystemSolution****
  
          This interface is used to represent a general solution to a
system of linear equations.

****LinearSystemSolver****
  
          This interface is used to represent classes that solve systems of linear
equations.

Classes in sbrt.kernel.math.algebra used by sbrt.kernel.fba

****SparseDoubleMatrix****
  
          This interface is used to represent sparse matrices whose
elements are strictly double precision numbers.

Classes in sbrt.kernel.math.algebra used by sbrt.kernel.fba.comp

****DoubleMatrix****
  
          This interface is used to represent matrices whose elements
are strictly double precision numbers.

****LinearSystemSolution****
  
          This interface is used to represent a general solution to a
system of linear equations.

****LinearSystemSolver****
  
          This interface is used to represent classes that solve systems of linear
equations.

****SparseDoubleMatrix****
  
          This interface is used to represent sparse matrices whose
elements are strictly double precision numbers.

Classes in sbrt.kernel.math.algebra used by sbrt.kernel.fba.proc

****DoubleMatrix****
  
          This interface is used to represent matrices whose elements
are strictly double precision numbers.

****IndexPair****
  
          This class is used to represent pairs of matrix indices.

****LinearSystemSolution****
  
          This interface is used to represent a general solution to a
system of linear equations.

****LinearSystemSolver****
  
          This interface is used to represent classes that solve systems of linear
equations.

****SparseDoubleMatrix****
  
          This interface is used to represent sparse matrices whose
elements are strictly double precision numbers.

Classes in sbrt.kernel.math.algebra used by sbrt.kernel.math.algebra

****AbstractMdMatrix****
  
          This abstract class is a skeleton implementation of
`DoubleMatrixBuilder`.

****AbstractSmdMatrix****
  
          This abstract class is a skeleton implementation of
`SmdMatrix`.

****DoubleMatrix****
  
          This interface is used to represent matrices whose elements
are strictly double precision numbers.

****DoubleMatrixBuilder****
  
          This interface is used to represent mutable matrices whose
elements are strictly double precision numbers.

****DoubleMatrixWrap****
  
          This class is used to wrap mutable matrices as an immutable
`DoubleMatrix`.

****HashMatrix****
  
          This class is a `java.util.LinkedHashMap` based implementation of `SmdMatrix`.

****IndexPair****
  
          This class is used to represent pairs of matrix indices.

****LinearSystemSolution****
  
          This interface is used to represent a general solution to a
system of linear equations.

****Matrix****
  
          This interface is used to represent matrices.

****MatrixBuilder****
  
          This interface is used to represent mutable matrices.

****MatrixWrap****
  
          This class is used to wrap mutable matrices as an immutable
matrix.

****RowOrTreeMatrix****
  
          This class is a `java.util.TreeMap` based implementation of `SmdMatrix`.

****SmdMatrix****
  
          This interface is used to represent sparse mutable matrices
containing strictly double precision numbers as elements.

****SparseDoubleMatrix****
  
          This interface is used to represent sparse matrices whose
elements are strictly double precision numbers.

****TreeMatrix****
  
          This class is a `java.util.TreeMap` based implementation of `SmdMatrix`.

Classes in sbrt.kernel.math.algebra used by sbrt.kernel.math.algebra.proc

****LinearSystemSolution****
  
          This interface is used to represent a general solution to a
system of linear equations.

****LinearSystemSolver****
  
          This interface is used to represent classes that solve systems of linear
equations.

Classes in sbrt.kernel.math.algebra used by sbrt.kernel.math.geom

****LinearSystemSolution****
  
          This interface is used to represent a general solution to a
system of linear equations.

Classes in sbrt.kernel.math.algebra used by sbrt.shell.io

****SparseDoubleMatrix****
  
          This interface is used to represent sparse matrices whose
elements are strictly double precision numbers.

Classes in sbrt.kernel.math.algebra used by sbrt.shell.mng.fba

****DoubleMatrix****
  
          This interface is used to represent matrices whose elements
are strictly double precision numbers.

****LinearSystemSolution****
  
          This interface is used to represent a general solution to a
system of linear equations.

Classes in sbrt.kernel.math.algebra used by sbrt.shell.text.math

****IndexPair****
  
          This class is used to represent pairs of matrix indices.

****MatrixElement****
  
          This class is used to represent matrix elements.

---


|  |  |  |
| --- | --- | --- |
| **Overview** \| **Package** \| Class \| **Use** \| **Tree** \| **Deprecated** \| **Index** \| **Help** | |  |
| PREV   NEXT | **FRAMES**    **NO FRAMES**     **All Classes** |  |


---
